# Determinants of health-related quality of life in pediatric epilepsy: the impact of treatment tolerability and epilepsy type

**DOI:** 10.3389/fneur.2026.1893873

**Published:** 2026-08-10

**Authors:** Ziad Zein, Dana Ayoub, Jana Baydoun, Nada El Eter, Bahia Chahine, Mohamad Rahal, Amal Al-Hajje, Mohammad Jammoul, Abdelrahman Albaba, Fatima Jaafar, Ahmad Beydoun

**Affiliations:** 1 School of Pharmacy, Lebanese International University, Beirut, Lebanon; 2 Clinical and Epidemiological Research Laboratory, Faculty of Pharmacy, Lebanese University, Beirut, Lebanon; 3 INSPECT-LB (Institut National de Santé Publique, d'Épidémiologie Clinique et de Toxicologie-Liban), Beirut, Lebanon; 4 Department of Neurology, American University of Beirut Medical Center, Beirut, Lebanon

**Keywords:** adverse effects, antiseizure medication, health-related quality-of-life, pediatric epilepsy, seizure frequency

## Abstract

**Background:**

Health-related quality-of-life (HRQOL) is a central outcome in pediatric epilepsy, yet the relative influence of disease severity versus treatment-related burden remains unclear. This study aimed to evaluate HRQOL and assess the respective contributions of clinical characteristics and treatment adverse events in children with epilepsy.

**Methods:**

A cross-sectional analysis of 101 children enrolled in a prospective cohort in Lebanon was conducted. HRQOL was assessed using the Arabic QOLCE-55, and treatment-related adverse effects were measured using the Liverpool Adverse Events Profile (LAEP). Multivariable linear regression models, including hierarchical domain-specific analyses, were used to identify factors independently associated with HRQOL.

**Results:**

HRQOL was moderately reduced, with a median QOLCE-55 score of 66 (IQR 55.68–79.41). The physical domain was the most affected (44.44; IQR 30.55–59.72), while the social domain was the least impaired (82.14; IQR 67.85–96.42). In multivariable analysis, higher adverse-effect burden was the strongest factor associated with poorer overall HRQOL (B = −0.97, 95% CI –1.28 to −0.67; *p* < 0.001), whereas most seizure-related variables were not significant after adjustment. Inclusion of LAEP substantially improved model performance (Δ*R*^2^ up to 0.310) and was associated with poorer HRQOL across all domains. Longer epilepsy duration remained associated with poorer physical HRQOL, and focal epilepsy with lower social and emotional scores.

**Conclusion:**

In this cohort, HRQOL was more strongly associated with treatment-related adverse effects than with most seizure-related variables. Treatment tolerability appears to represent an important and modifiable correlate of well-being. Routine assessment of adverse effects may enhance patient-centered care.

## 1 Introduction

Epilepsy is among the most common chronic neurological disorders of childhood and adolescence, with consequences that extend well beyond the occurrence of seizures (1). In addition to neurological morbidity, children with epilepsy frequently experience academic underachievement, limitations in daily activities, behavioral and psychiatric comorbidities, and social and emotional difficulties (2–5). These challenges may influence not only the child’s development and functioning but also family dynamics and long-term psychosocial outcomes.

For these reasons, health-related quality-of-life (HRQOL) has emerged as an important patient-centered outcome in pediatric epilepsy. HRQOL captures the broader functional, cognitive, emotional, and social impact of the disease and its treatment, complementing traditional clinical measures such as seizure frequency. Studies have consistently shown that children with epilepsy have lower HRQOL compared with healthy peers and even with children affected by other chronic illnesses, particularly in cognitive, emotional, and school-related domains (4). These observations suggest that the burden of epilepsy cannot be fully understood through seizure counts alone.

A growing body of literature indicates that HRQOL in childhood epilepsy is influenced by multiple clinical and social determinants. Seizure-related variables, including frequency, severity, and seizure-free duration, have been repeatedly linked to poorer outcomes (5–12) as has longer epilepsy duration (12). Socioeconomic and caregiver-related factors, such as parental education and employment status, have also been implicated (11). Treatment-related variables represent another important dimension. Several studies have reported associations between polytherapy and reduced quality-of-life (5, 11, 13, 14), and an increasing number of reports suggest that adverse effects of ASM may play a particularly important role (9, 10, 15). Drug tolerability refers to the degree to which the overt adverse effects of a treatment can be tolerated by the patient (16). However, the relative contribution of seizure burden versus treatment tolerability remains incompletely understood. Notably, studies in adults have shown that once medication-related adverse effects are taken into account, the apparent impact of seizure frequency on quality-of-life may diminish substantially (6), raising the possibility that treatment burden may be a more direct contributor to functional impairment.

Despite growing interest in this area, data from pediatric populations in low and middle-income settings remain limited, and few studies have examined HRQOL using epilepsy-specific instruments while simultaneously evaluating treatment tolerability. Understanding these relationships is particularly important in children, in whom cognitive development, school performance, and social participation may be especially sensitive to both disease chronicity and medication effects.

In this context, we conducted a cross-sectional study nested within an ongoing prospective cohort of children with epilepsy in Lebanon. The aims of this study were: (1) to characterize HRQOL in children and adolescents with epilepsy using the validated Arabic version of the QOLCE-55 (17), and (2) to identify sociodemographic, caregiver-related, clinical, and treatment-related factors, including medication adverse events, associated with HRQOL in this population.

## 2 Methods

### 2.1 Study design

This was a cross-sectional study conducted at the American University of Beirut Medical center (AUBMC), Lebanon. Participants were selected from an ongoing prospective multicenter cohort study entitled *“Newly Onset Epilepsy in Lebanon,”* which enrolls patients at the time of their epilepsy diagnosis through a nationwide referral network of neurologists practicing across the six Lebanese governorates. Referred patients undergo a comprehensive clinical evaluation and standardized diagnostic workup at AUBMC. The design and methodology of this cohort have been described previously (18).

For the present study, a random sample of children was selected from the cohort, and their parents or primary caregivers were administered quality-of-life questionnaires between March 2024 and July 2024. At the time of questionnaire administration, each child’s medical record was reviewed in detail and updated to ensure accuracy of clinical, seizure-related, and treatment-related variables.

### 2.2 Study participants

Children were eligible for inclusion if they were between 5 and 18 years of age, had a confirmed diagnosis of epilepsy for at least 6 months, and were receiving ASM therapy at the time of assessment. Children were excluded if they had a documented history of intellectual disability, global developmental delay, autism spectrum disorder, or other significant behavioral or neurodevelopmental disorders that could independently affect HRQOL or require the use of different quality-of-life instruments. A total of 583 children in the parent prospective cohort met the study eligibility criteria. Eligible participants were randomly assigned to four recruitment lists, which were distributed among four research fellows who contacted families by telephone according to their assigned list until the target sample size was reached. Overall, 143 telephone calls were made, 117 families were successfully contacted, and 101 agreed to participate (participation rate among families reached: 86.3%).

### 2.3 Outcome measure: HRQOL

HRQOL was assessed using the Arabic version of the Quality-of-Life in Childhood Epilepsy questionnaire (QOLCE-55), a parent-reported instrument designed for children aged 4 to 18 years (19). The reliability and validity of the Arabic version of the QOLCE-55 were previously established in a pediatric epilepsy population (17).

The QOLCE-55 consists of 55 items grouped into 16 subscales encompassing four core domains of HRQOL: cognitive functioning (22 items), emotional functioning (17 items), social functioning (7 items), and physical functioning (9 items). Items are rated on a five-point Likert scale ranging from 0 (“very often”) to 4 (“never”) (20). Negatively worded statements are reverse-coded so that higher scores consistently reflect better HRQOL. Raw item scores were transformed to a 0–100 scale (0, 25, 50, 75, and 100), and mean scores were calculated for each subscale. Domain scores were derived by averaging relevant subscales, and the total QOLCE-55 score was calculated as the unweighted mean of the four domain scores.

### 2.4 Study variables

- Demographic variables included age, gender, origin and place of residence, primary caregiver, educational status of the primary caregiver, and family income.
- Disease-related variables included age at seizure onset, duration of epilepsy, seizure types, number of seizure types, epilepsy syndrome classification, seizure timing (diurnal, nocturnal, or mixed), seizure frequency in the preceding 3 months, time since last seizure and presence of comorbidities.
- Treatment-related variables included duration on ASM therapy, number and types of current ASM, history of ASM discontinuation due to lack of efficacy (failure of ASM due to seizure recurrence) or poor tolerability (clinician-reported failure of an ASM due to intolerable adverse effects), and treatment regimen (monotherapy vs. polytherapy; ≥2 ASM).
- Treatment-related adverse-effect burden were measured using the Liverpool Adverse Events Profile (LAEP) scale, an epilepsy-specific questionnaire developed and validated by Baker et al. (21). The LAEP consists of 19 items evaluating patient or caregiver-perceived adverse effects of ASM. Each item is rated on a four-point Likert scale from 1 (“never a problem”) to 4 (“often or always a problem”), yielding a total score ranging from 19 to 76, with higher scores indicating a greater burden of adverse effects. The LAEP has already been validated in Arabic language (22) and has been previously completed by parents to assess caregiver-reported adverse effects (23).
- Domain-specific QOLCE-55 scores (Cognitive, Physical, Social, Emotional) were analyzed as secondary outcomes.

### 2.5 Ethical approval and consent

This study was approved by the Institutional Review Board of AUBMC. Written informed consent was obtained from a parent or legal guardian for all participating children prior to enrollment.

### 2.6 Sample size calculation

Sample size estimation was performed using G*Power version 3.1.9.7 for an F-test based on a fixed multiple linear regression model (*R*^2^ deviation from zero). Assuming an anticipated effect size of Cohen’s *f*^2^ = 0.28 (6), a two-sided significance level of *α* = 0.05, 80% statistical power, and 15 candidate predictors in the multivariable model, the minimum required sample size was 81 participants.

### 2.7 Statistical analysis

Descriptive statistics were used to summarize the demographic, clinical and treatment characteristics. Categorical variables were reported as frequencies and percentages, while continuous variables were expressed as means ±standard deviations or medians with interquartile ranges, as appropriate. Internal consistency of the Arabic versions of the LAEP and the QOLCE-55 was assessed using Cronbach’s *α*.

Bivariable associations with the total QOLCE-55 score were evaluated using the Student’s t-test or one-way ANOVA for categorical variables, and the Spearman rank correlation for continuous variables. Variables associated with the total QOLCE-55 score at *p* < 0.20 in bivariable analyses, along with clinically relevant covariates selected *a priori*, were included in the multivariable linear regression model. In contrast, for domain-specific outcomes, multivariable models were constructed using a prespecified hierarchical approach based on clinical reasoning rather than bivariable screening. The order of entry of variables into hierarchical models was defined *a priori* based on clinical plausibility and temporal precedence.

For each domain, disease-related characteristics (epilepsy duration, time since last seizure, seizure frequency, and epilepsy type) were entered in the first block. Treatment-related adverse-effect burden, as measured by the LAEP score, was entered in the second block. Current treatment regimen (monotherapy versus polytherapy) was entered in the final block. LAEP was conceptualized as a competing explanatory variable reflecting the child’s current treatment-related adverse-effect burden rather than as a conventional confounder. Accordingly, the hierarchical models were designed to assess the contribution of adverse-effect burden to HRQOL beyond disease-related characteristics and treatment regimen.

Variables reflecting cumulative treatment exposure (total number of ASM ever received) were not entered simultaneously with LAEP to avoid collinearity and over-adjustment, as LAEP captures the current patient-perceived burden of treatment.

Missing data were handled using complete-case analysis. No imputation procedures were performed. Missing observations were confined to the primary caregiver education variable and did not involve the primary exposure (LAEP) or outcome (QOLCE-55).

Model performance was assessed using adjusted *R*^2^ values. Assumptions of linear regression were verified by inspection of residual distributions, evaluation of influential observations using Cook’s distance, and assessment of multicollinearity using variance inflation factors.

A two-sided *p*-value < 0.05 was considered statistically significant. All statistical analyses were performed using SPSS, version 26.

## 3 Results

### 3.1 Sociodemographic and caregiver related characteristics

A total of 101 children with epilepsy were included. The age at assessment ranged from 6.4 to 17.8 years, with a mean age of 12.8 ± 3.2 years. Fifty-four children (53.5%) were male, and the majority were of Lebanese nationality (86.8%). The primary caregiver was the mother in most cases (94%). Thirty-nine percent of primary caregivers had attained a university degree or higher, and more than half of families reported a monthly income below USD 500 (58.4%). Sociodemographic characteristics are summarized in Table 1.

**Table 1:** Demographic, clinical, and treatment characteristics of the study population.

Variable	Mean (±SD)	Range	Median (IQR)
Age (years)	12.8 ± 3.2	6.4–17.8	13.5 (9.9–15.1)
Age at epilepsy onset (years)	7.7 ± 3.8	0.4–15.9	8.3 (4.2–10.6)
Epilepsy duration (years)	5.0 ± 2.3	0.6–17.6	5.1 (4.1–6.1)

Variable	*N* (%)
Gender	
Male	54 (53.5)
Female	47 (46.5)
Nationality	
Lebanese	86 (86.8)
Other	15 (14.2)
Primary caregiver	
Mother	95 (94.0)
Father	6 (6.0)
Primary caregiver education (missing = 16)	
Primary/elementary	30 (35.3)
Secondary	22 (25.8)
University/higher education	33 (38.8)
Monthly family income	
<500$	59 (58.4)
500$ < 1,000$	34 (33.7)
>1,000$	8 (7.9)
Epilepsy group	
Genetic generalized epilepsy	32 (31.7)
Self-limited focal epilepsy	22 (21.8)
Focal epilepsy	47 (46.5)
Seizure type	
FIAS	40 (39.6)
FBTC	32 (31.7)
FAS	18 (17.8)
GOTC	26 (25.7)
Absence Seizures	9 (8.9)
Myoclonic Seizures	8 (7.9)
Other*	1 (1.0)
Number of seizure types	
One type	69 (68.3)
≥Two types	32 (31.7)
Time of seizure occurrence	
Diurnal	55 (54.4)
Nocturnal	22 (21.8)
Mixed	24 (23.8)
Seizure frequency (past 3 months)	
Seizure free	73 (72.3)
Less than once per month	6 (5.9)
1–3 seizures per month	15 (14.9)
1–3 seizures per week	4 (4.0)
Daily	3 (3.0)
Number of ASM (current)	
One	77 (76.2)
Two	18 (17.8)
≥Three	6 (6.0)
ASM type (current)	
Levetiracetam	38 (37.6)
Valproate	34 (33.7)
Lacosamide	18 (17.8)
Oxcarbazepine	12 (11.9)
Carbamazepine	9 (8.9)
Clonazepam	8 (7.9)
Other	12 (12.0)

SD, standard deviation; IQR, interquartile range. Other ASM: topiramate in 5 children (5.0%), lamotrigine in 3 children (3.0%), ethosuxmide in 2 children (2.0%), and phenobarbital and clobazam one child each (1.0%). FIAS, focal impaired awareness seizure; FBTC, focal to bilateral tonic–clonic seizure; FAS: focal aware seizure; GOTC, generalized onset tonic–clonic seizure.

### 3.2 Disease characteristics

The age at seizure onset ranged from 4.8 months to 15.9 years (median 8.3 years, IQR 4.2–10.6) and the duration of epilepsy at the time of assessment ranged from 0.6 to 17.6 years (median 5.1 years, IQR 4.1–6.1). Only four children (4%) had documented comorbidities. Focal epilepsy was the most common epilepsy type, diagnosed in 47 children (46.5%), followed by generalized genetic epilepsies (GGEs) in 32 (31.7%), and self-limited focal epilepsies (SeLFEs) in 22 (21.8%).

The most frequent seizure types were focal impaired awareness seizures (FIAS) (39.6%) and focal to bilateral tonic–clonic seizures (FBTC) (31.7%). Most children experienced a single seizure type (68.3%). Seizures occurred predominantly during wakefulness (54.5%), while 21.8% had exclusively nocturnal seizures and 23.8% had mixed patterns. Over the preceding 3 months, most children were seizure-free (72.3%). Seizure frequency was less than once per month in 5.9%, one to three times per month in 14.9%, one to three times per week in 4.0%, and daily in 3.0% of participants (Table 1).

### 3.3 Treatment characteristics

At the time of assessment, most children were on ASM monotherapy (76.2%), while 17.8% were on dual therapy and 6.0% were receiving three or more ASM. The most frequently prescribed ASM were levetiracetam (37.6%) and valproate (33.7%), followed by lacosamide (17.8%) and oxcarbazepine (11.9%). Over the course of treatment, 54.5% of children received a single ASM, 23.8% were exposed to two ASM, and 21.9% to three or more ASM. Seventeen children (16.8%) had discontinued one ASM due to lack of efficacy, while four (4%) had failed two ASM for the same reason. Fifteen children (14.8%) had discontinued at least one ASM because of intolerable adverse effects (Table 1).

### 3.4 Liverpool adverse events profile (LAEP)

**Figure 1:**
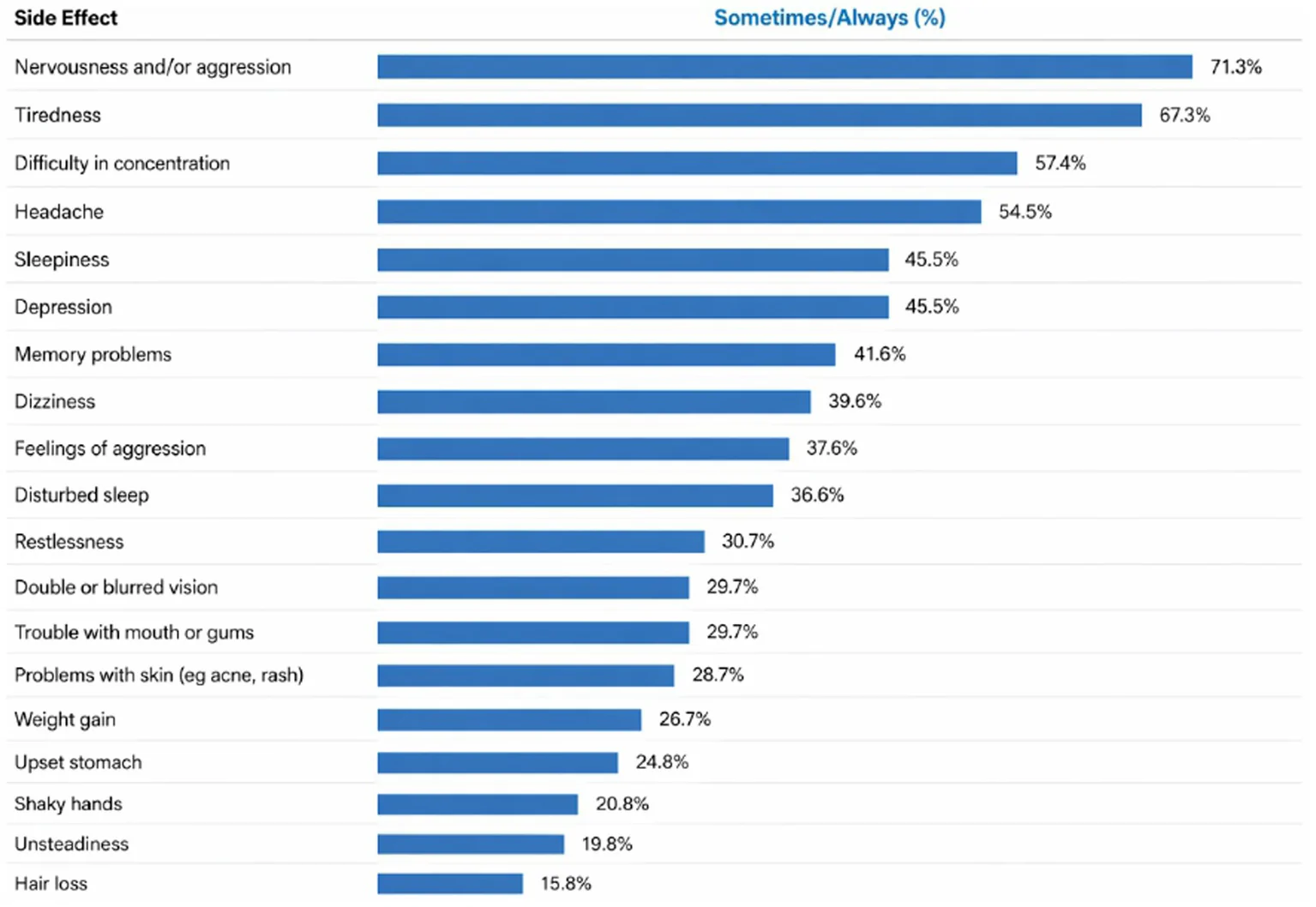
Prevalence of adverse effects reported “sometimes” or “always” by caregivers of children with epilepsy using the liverpool adverse events profile (LAEP).

The mean LAEP score was 38.75 (SD = 9.42), with a median of 40 (IQR 32.5–45) and a range of 19 to 58. The most frequently reported adverse events, occurring either sometimes or often, were nervousness and/or aggression (71.3%), tiredness (67.3%), difficulty concentrating (57.4%), and headaches (54.4%). Shaky hands (20.8%), unsteadiness (19.8%), and hair loss (15.8%) were the least frequently reported (Figure 1). The Arabic version of the LAEP demonstrated strong internal consistency (Cronbach’s *α* = 0.867).

### 3.5 The quality of life in childhood epilepsy questionnaire (QOLCE-55) score

The median QOLCE-55 score was 65.05 (IQR 55.68–79.41). Among the four domains, physical functioning was most affected, with the lowest median score (44.44, IQR 30.55–59.72). Cognitive and emotional domains showed intermediate impairment, with median scores of 70.45 (IQR 51.70–86.36) and 70.58 (IQR 59.55–81.61), respectively. Social functioning was relatively preserved, with the highest median score (82.14, IQR 67.85–96.42) (Table 2). The Arabic QOLCE-55 showed excellent internal consistency (Cronbach’s *α* = 0.963).

**Table 2:** Quality of life in childhood epilepsy questionnaire (QOLCE-55) total and sub-domain scores.

Variables	Range	Mean ± SD	Median (IQR)
Quality of life			
Cognitive score	21.59–100	67.99 ± 21.33	70.45 (51.70–86.36)
Emotional score	26.47–98.53	68.66 ± 16.86	70.58 (59.55–81.61)
Social score	25–100	77.86 ± 20.34	82.14 (67.85–96.42)
Physical score	11.11–97.22	45.68 ± 19.18	44.44 (30.55–59.72)
Total QOLCE-55 score	26.29–95.63	65.05 ± 15.69	66 (55.68–79.41)

SD, standard deviation; IQR, interquartile range; QOLCE-55, Quality of life in childhood epilepsy questionnaire-55 item.

### 3.6 Bivariable associations with the QOLCE-55 score

None of the demographic or caregiver-related variables, including monthly family income, were significantly associated with total QOLCE-55 score in bivariable analyses. Longer epilepsy duration was modestly associated with poorer QOLCE-55 score (*r* = −0.207, *p* = 0.039), whereas longer time since last seizure was strongly associated with better quality-of-life (*r* = 0.456, *p* < 0.001).

Children who were seizure free during the preceding three months had the highest QOLCE-55 score (68.34 ± 14.94), with progressively lower scores observed as seizure frequency increased (*p* < 0.001) (Table 3). Children receiving monotherapy had significantly higher QOLCE-55 scores compared with those on polytherapy (66.85 ± 15.60 vs. 59.27 ± 14.87; *p* = 0.038).

**Table 3:** Bivariable associations between QOLCE-55 score and socio-demographic, caregiver, clinical, and treatment characteristics.

Variables	QOLCE-55 score		Spearman’s correlation (QOLCE-55)	
	Mean ± SD	*p*-value	*r*	*p*-value
Demographic variables				
Age	—	—	0.066	0.523
Gender				
Male	65.56 ± 17.05	0.726	—	—
Female	64.45 ± 14.13		—	—
Family income				
Less than 500$	65.47 ± 15.27	0.383	—	—
500$—1000$	62.85 ± 16.95		—	—
1,000$—3000$	71.20 ± 12.75		—	—
Caregiver related variables				
Primary caregiver				
Mother	64.77 ± 16	0.480	—	—
Father	69.46 ± 9.29		—	—
Primary caregiver education				
Primary/elementary	59.51 ± 17.07	0.445	—	—
Secondary	64.27 ± 19.92		—	—
University/higher education	63.19 ± 17.23		—	—
Disease related variables				
Age at onset of epilepsy (years)	—	—	0.160	0.114
Epilepsy duration (years)	—	—	−0.207	**0.039**
Time since last seizure (months)	—	—	0.456	**< 0.001**
Seizure type				
Focal Seizures	63.55 ± 16.79	0.162	—	—
Generalized Seizures	68.27 ± 12.66		—	—
Number of seizure types				
One type	63.96 ± 16.08	0.308	—	—
Two or more types	67.39 ± 14.79		—	—
Time of seizure occurrence				
Nocturnal	62.34 ± 16.06	0.368	—	—
Diurnal	67.07 ± 15.5		—	—
Mixed	62.89 ± 15.79		—	—
Seizure Frequency/past 3 months				
None	68.34 ± 14.94	**< 0.001**	—	—
Less than one per month	68.34 ± 9.99		—	—
1–3 seizures per month	55.46 ± 13.28		—	—
1–3 seizures per week	45.81 ± 15.37		—	—
Daily	52.01 ± 18.2		—	—
Epilepsy group				
GGE	68.27 ± 12.66	**0.039**	—	—
SeLFE	69.37 ± 14.81		—	—
Focal Epilepsy	60.83 ± 17.12		—	—
Comorbidities				
Yes	63.4 ± 12.99	0.832	—	—
No	65.11 ± 15.55		—	—
Treatment related variables				
Number of current ASMs				
Monotherapy	66.85 ± 15.60	**0.038**		
Polytherapy	59.27 ± 14.87			
Liverpool adverse events profile score	—	—	−0.730	**< 0.001**
Total No. of ASMs ever received	—	—	−0.231	**0.020**
No. of ASMs failed/lack of tolerability	—	—	−0.175	**0.082**
No. of ASMs failed/lack of efficacy			−0.008	0.940

QOLCE-55, Quality of life in childhood epilepsy questionnaire 55 item; ASM, antiseizure medication; SD, standard deviation; GGE, Genetic generalized epilepsy; SeLFE, Self-limited focal epilepsy. *P*-values in bold indicate statistically significant findings (*P* < 0.05).

**Figure 2:**
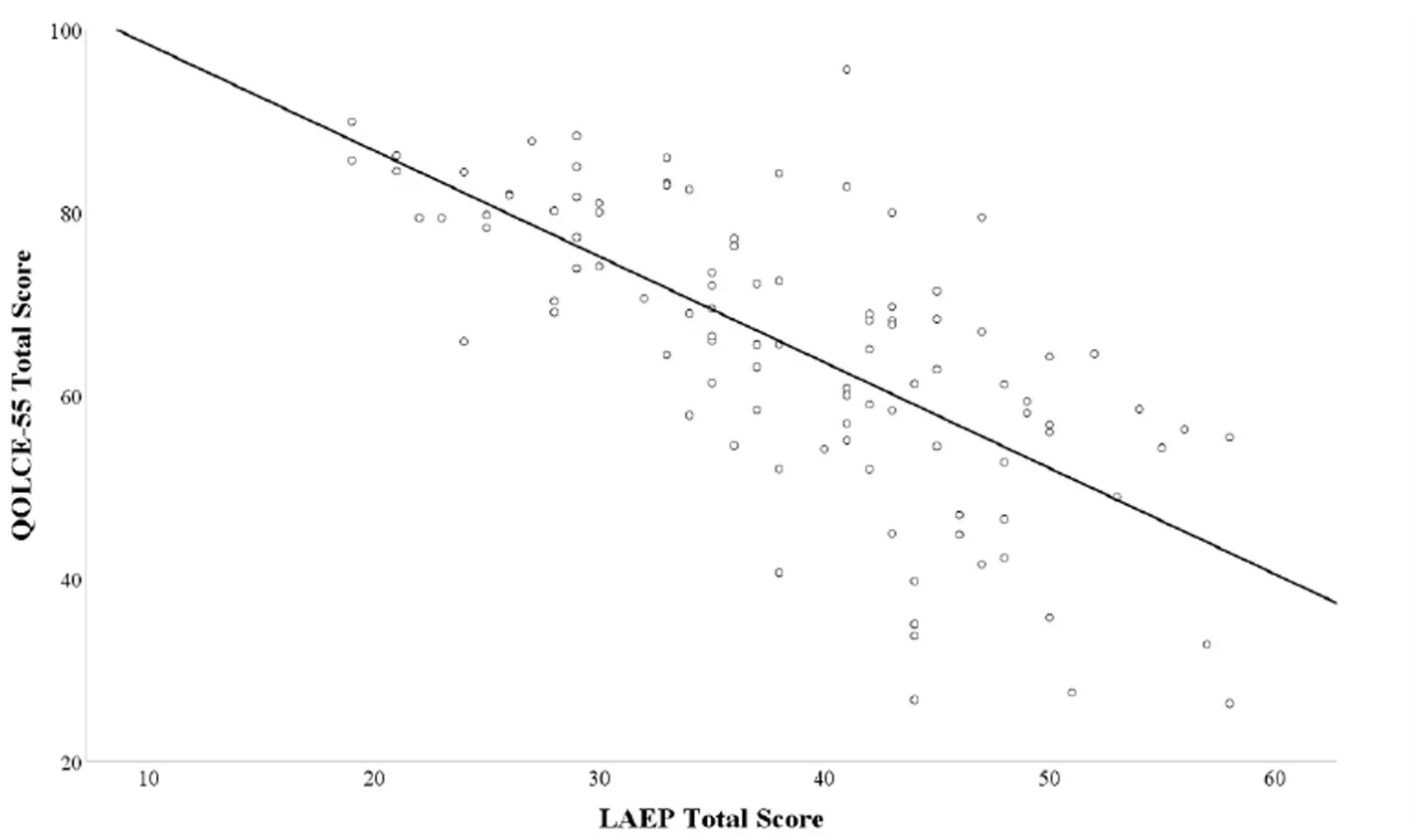
Association between LAEP total score and total QOLCE-55 score in children with epilepsy. LAEP, Liverpool adverse events profile; QOLCE-55, Quality of life in childhood epilepsy questionnaire-55.

Total QOLCE-55 scores showed a strong negative correlation with LAEP scores (*r* = −0.73, *p* < 0.001). This relationship is illustrated in Figure 2, which shows a clear inverse association between LAEP scores and total QOLCE-55 scores. Lower quality-of-life was also associated with a higher total number of ASM ever received (*r* = −0.231, *p* = 0.020), while the number of ASM discontinued due to poor tolerability showed a weaker, non-significant trend (*r* = −0.175, *p* = 0.082) (Table 3).

### 3.7 Bivariable analyses with domain-specific QOLCE-55 scores

Given the observed differences in overall domain scores, domain-specific bivariable analyses were conducted to identify factors associated with each quality-of-life dimension.

Higher LAEP scores were consistently associated with poorer quality-of-life across all domains, including cognitive (*ρ* = −0.645, *p* < 0.001), physical (*ρ* = −0.404, *p* < 0.001), social (*ρ* = −0.683, *p* < 0.001), and emotional (*ρ* = −0.595, p < 0.001).

Longer epilepsy duration was associated with lower cognitive (*ρ* = −0.245, *p* = 0.014) and physical scores (*ρ* = −0.228, *p* = 0.023), but was not significantly associated with social (*ρ* = −0.160, *p* = 0.112) or emotional scores (*ρ* = 0.045, *p* = 0.657). In contrast, longer time since the last seizure was associated with higher scores across all domains: cognitive (*ρ* = 0.370, *p* < 0.001), physical (*ρ* = 0.295, *p* = 0.003), social (*ρ* = 0.373, *p* < 0.001), and emotional (*ρ* = 0.425, *p* < 0.001).

Children receiving polytherapy had lower cognitive scores compared with those receiving monotherapy (mean difference = 10.9 points, *p* = 0.029), while no significant differences were observed for the other domains. Similarly, a higher total number of ASM ever received was associated with lower cognitive scores (*ρ* = −0.280, *p* = 0.005) and showed a trend toward lower physical scores (*ρ* = −0.180, *p* = 0.071), with no significant associations for social or emotional domains.

Emotional scores differed across epilepsy types (*p* = 0.029), with children with focal epilepsy showing lower scores compared with those with SeLFEs, whereas cognitive, physical, and social scores did not significantly differ by epilepsy type.

### 3.8 Multivariable analysis

#### 3.8.1 Total QOLCE-55 score

**Figure 3:**
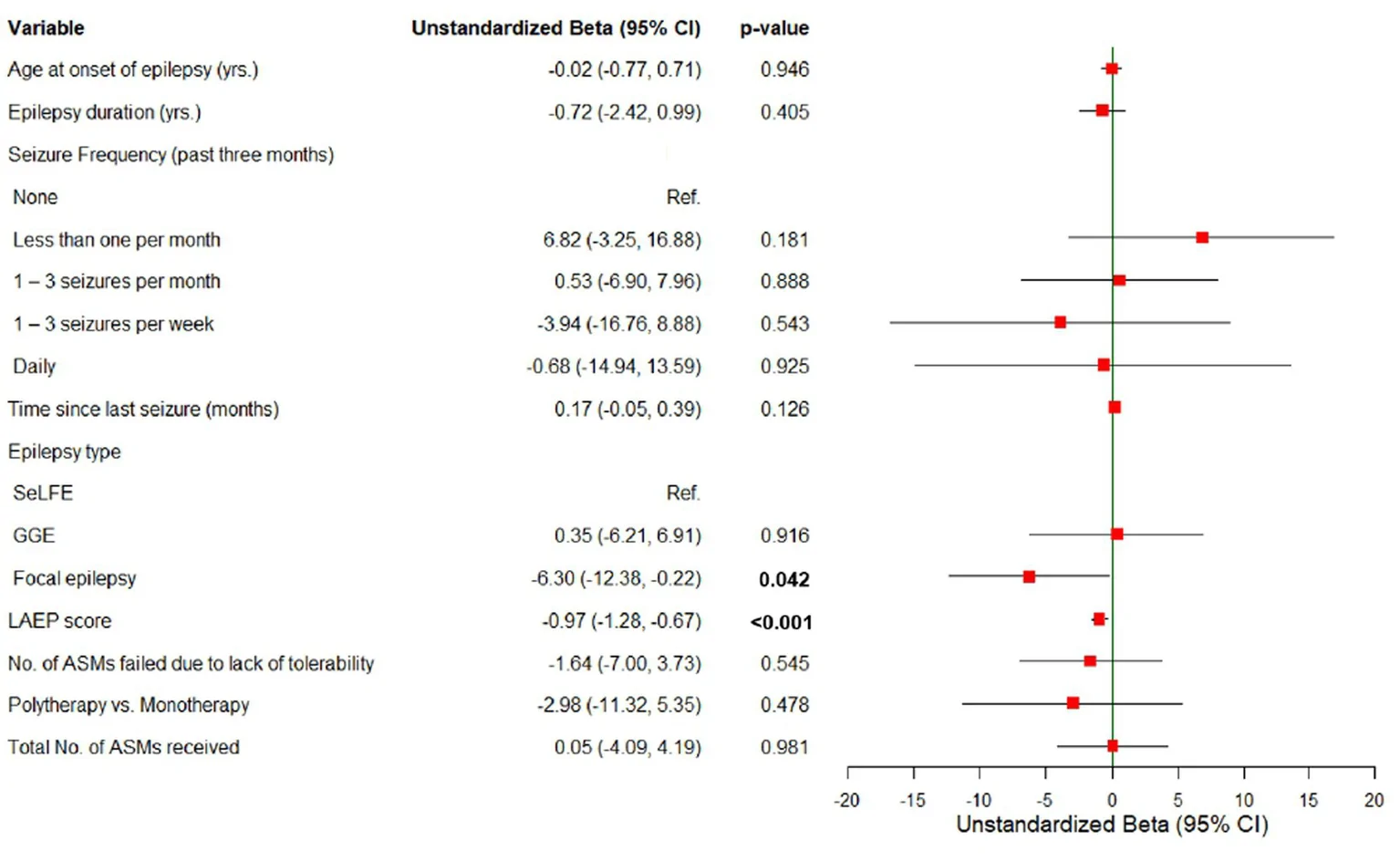
Forest plot of multivariable linear regression results of factors associated with QOLCE-55 scores. GGE, generalized genetic epilepsy; SeLFE, self-limited focal epilepsy; LAEP, Liverpool adverse events profile; ASM, antiseizure medication.

In multivariable linear regression analysis of the total QOLCE-55 score (Figure 3), most clinical variables, including age at seizure onset, epilepsy duration, seizure frequency, and time since last seizure, were not independently associated with quality-of-life after adjustment. Epilepsy type emerged as a significant predictor, with children with focal epilepsy having lower QOLCE-55 scores compared with those with SeLFes (B = −6.30, 95% CI –12.38 to −0.22; *p* = 0.042). Higher LAEP scores were strongly associated with poorer quality-of-life (B = −0.97, 95% CI –1.28 to −0.67; *p* < 0.001). Other treatment-related variables did not retain independent associations. The final model explained a substantial proportion of variance in total QOLCE-55 scores (adjusted *R*^2^ = 0.507).

#### 3.8.2 QOLCE-55 sub-domains

In hierarchical multivariable analyses across the QOLCE-55 sub-domains, disease-related clinical variables (epilepsy duration, time since last seizure, seizure frequency, and epilepsy type) explained a modest proportion of variance in scores. Addition of LAEP scores substantially improved model performance across all domains, with increases in explained variance of Δ*R*^2^ = 0.242 for the cognitive domain, Δ*R*^2^ = 0.042 for the physical domain, Δ*R*^2^ = 0.310 for the social domain, and Δ*R*^2^ = 0.195 for the emotional domain.

#### 3.8.3 Cognitive domain

In the fully adjusted model, only higher adverse-effect burden (higher LAEP score) was significantly associated with poorer cognitive quality-of-life (B = −1.28, 95% CI –1.69 to −0.87; *p* < 0.001). Other clinical variables were no longer significant after adjustment for LAEP. Treatment regimen (monotherapy vs. polytherapy) did not contribute additional explanatory value.

#### 3.8.4 Physical domain

Only higher LAEP scores (B = −0.51, 95% CI –0.95 to −0.06; *p* = 0.026) and longer epilepsy duration (B = −1.83, 95% CI –3.44 to −0.21; *p* = 0.027) were independently associated with poorer physical quality-of-life. Other disease-related variables became non-significant, and treatment regimen did not significantly improve model fit.

#### 3.8.5 Social domain

Higher LAEP scores (B = −1.27, 95% CI –1.69 to −0.88; *p* < 0.001) and focal epilepsy vs. SeLFEs (B = −8.45, 95% CI –16.55 to −0.35; *p* = 0.047) were associated with poorer social quality-of-life scores. Other clinical variables were not significant, and treatment regimen did not improve model fit.

#### 3.8.6 Emotional domain

Higher LAEP scores remained independently associated with poorer emotional scores (B = −0.94, 95% CI –1.27 to −0.68; *p* < 0.001). Focal epilepsy, compared with SeLFEs, was also associated with lower emotional scores (B = −8.84, 95% CI –15.82 to −1.87; *p* = 0.014). Other clinical variables were no longer significant after adjustment and treatment regimen did not improve model fit.

Detailed multivariable results for subdomains are provided in Supplementary Table S1. For all multivariable models, assumptions of linear regression were met, with approximately normally distributed residuals, no influential observations (Cook’s distance < 1), and low multicollinearity (variance inflation factors < 2).

## 4 Discussion

This study examined HRQOL in a well-characterized cohort of children with epilepsy using validated Arabic versions of the QOLCE-55 and LAEP. Several consistent observations emerged. Overall quality-of-life was moderately reduced, with the Physical domain most affected and the Social domain relatively preserved. Across global and domain-specific analyses, treatment-related adverse effects showed the strongest and most consistent associations with HRQOL, whereas most clinical and sociodemographic variables lost significance after adjustment. Epilepsy type remained relevant, with focal epilepsies associated with poorer overall quality-of-life, and epilepsy duration retained an independent association with the Physical domain.

The magnitude of HRQOL impairment observed in our cohort is comparable to that reported in pediatric epilepsy populations from high-income settings, where moderate reductions in quality-of-life have been consistently documented across cognitive, physical, and psychosocial domains (13, 24, 25). This similarity, despite differences in healthcare resources and socioeconomic context, suggests that the burden of epilepsy on daily functioning is substantial and cannot be understood solely in terms of seizure counts, but rather reflects the combined influence of treatment-related symptoms and underlying disease characteristics.

A distinctive feature of our data was the pattern of domain involvement. The Physical domain was most affected, followed by the Cognitive and Emotional domains, with relative preservation of Social functioning. Prior studies have not been uniform in identifying which domain is most vulnerable. Some pediatric cohorts have emphasized school performance or psychosocial difficulties (5, 26, 27) whereas others have highlighted physical limitations related to fatigue, reduced activity tolerance, and medication-related symptoms (28, 29). In this cohort, the predominance of physical impairment likely reflects the cumulative burden of treatment-related complaints, particularly nervousness/aggression, tiredness, and concentration difficulties, which were among the most frequently reported symptoms. The relatively preserved Social domain may reflect strong family cohesion and close caregiver involvement, which can buffer social participation even when physical stamina or cognitive efficiency are reduced.

Regional studies provide useful context for interpreting these findings. An Omani cohort similarly reported reduced HRQOL but identified seizure frequency, age at onset, treatment regimen, and socioeconomic factors as important determinants, whereas a cohort from Marrakesh highlighted seizure frequency and polytherapy as the principal predictors of impairment (28, 30). In contrast, sociodemographic variables were not independently associated with HRQOL in our study, and the apparent effects of seizure frequency and treatment regimen were substantially attenuated after accounting for treatment-related adverse effects. Differences in measurement instruments and healthcare context may partly explain these findings. Unlike the Marrakesh study, our analysis incorporated the LAEP, allowing direct quantification of treatment tolerability, which may explain why adverse-effect burden emerged as the dominant correlate of HRQOL.

The most consistent finding across analyses was the strong inverse association between LAEP scores and HRQOL. Higher adverse-effect burden remained independently associated with poorer overall HRQOL and all four domains after multivariable adjustment. Although some overlap exists between symptoms captured by the LAEP and domains assessed by the QOLCE-55, these symptoms represent clinically meaningful experiences that directly influence children’s daily functioning and perceived quality of life.

These findings are consistent with adult studies demonstrating that treatment-related adverse effects are more closely associated with HRQOL than seizure frequency and that systematic assessment of adverse effects can improve patient-reported outcomes (31). Our findings extend these observations to children. Similar associations have also been described in pediatric cohorts, where fatigue, irritability, and reduced attention adversely affect school performance and daily functioning (9, 10, 15). Together, these data emphasize that treatment tolerability deserves routine consideration alongside seizure control when managing pediatric epilepsy.

Epilepsy type also played an independent role. Children with focal epilepsies had lower overall HRQOL than those with SeLFEs and showed poorer outcomes in both emotional and social domains. This is consistent with reports that focal epilepsies of structural or unknown etiology may be associated with greater cognitive and behavioral comorbidity and may require more prolonged treatment (32, 33). A systematic review focusing on focal epilepsy similarly identified cognitive difficulties, mood symptoms, and treatment burden as recurrent contributors to reduced HRQOL (34).

Interestingly, seizure frequency and time since last seizure were not independent predictors once other variables were considered. This does not diminish the importance of seizure control but suggests that, in this cohort, its association with HRQOL may be partly reflected through treatment-related symptom burden. In daily clinical practice, a child who is seizure-free but experiencing fatigue, irritability, or cognitive slowing may still have substantial functional limitations.

These findings should also be interpreted in the context of the study population. More than 70% of children in our study were seizure-free during the preceding 3 months, most were receiving monotherapy, and comorbidities were uncommon. Consequently, the variability of seizure-related burden was relatively limited, which may have attenuated the independent associations between seizure-related variables and HRQOL. Different relationships may be observed in cohorts with a higher prevalence of ongoing seizures or drug-resistant epilepsy.

Finally, LAEP was conceptualized as a competing explanatory variable reflecting the child’s current treatment-related symptom burden rather than as a conventional confounder. Accordingly, attenuation of the associations between seizure-related variables, treatment intensity, and HRQOL after inclusion of the LAEP should not be interpreted as evidence that these factors are clinically unimportant. Rather, it suggests that part of their association with HRQOL may be reflected through the child’s current adverse-effect burden.

No independent associations were observed between HRQOL and sociodemographic variables, including caregiver education and family income. Previous studies have reported mixed findings (5, 8, 11, 26, 35). The absence of such associations in our cohort may reflect relatively homogeneous access to specialized care and strong caregiver involvement across socioeconomic strata.

This study has several strengths. Participants were randomly selected from a multicenter prospective cohort with detailed clinical documentation. The use of validated epilepsy-specific instruments enabled structured assessment of both HRQOL and treatment-related adverse effects in an Arabic-speaking population, an area where data remain limited. In addition, the inclusion of domain-specific analyses across all four QOLCE-55 domains provided a more comprehensive understanding of how different aspects of epilepsy and its treatment influence daily functioning.

Several limitations should be acknowledged. The cross-sectional design precludes causal inference, and HRQOL was assessed through caregiver reports, which may differ from child self-perception. In addition, some treatment-related symptoms assessed by the LAEP, such as fatigue, cognitive difficulties, and mood disturbances, overlap conceptually with domains captured by the QOLCE-55. Consequently, part of the observed association between adverse-effect burden and HRQOL may reflect shared constructs measured by both instruments. Children with developmental delay or autism were excluded because different assessment tools are required in these populations, and our findings may therefore not generalize to children with more complex neurodevelopmental profiles. In addition, the predominance of relatively well-controlled epilepsy in this cohort, as reflected by high rates of seizure freedom and monotherapy, may have attenuated the independent contribution of seizure-related variables and may limit generalizability to more refractory pediatric epilepsy populations. Finally, most participants were recruited from a tertiary center, which may limit generalizability but also ensured consistent evaluation and follow-up.

From a clinical perspective, these findings underscore the need to look beyond seizure counts alone when managing pediatric epilepsy. Adverse effects such as fatigue, irritability, and difficulty concentrating appear to have a direct and measurable impact across cognitive, physical, emotional, and social functioning. Routine assessment of treatment tolerability should therefore be an integral part of follow-up, and therapeutic decisions should balance seizure control with preservation of cognitive and physical well-being.

In conclusion, HRQOL in children with epilepsy appears to be shaped not solely by seizure-related variables, but by the combined influence of epilepsy type, disease chronicity, and the day-to-day burden of treatment. The strong and consistent association between adverse-effect burden and outcomes across all domains highlights treatment tolerability as a central clinical target and an important correlate of patient well-being. These observations support a more comprehensive model of care in which systematic monitoring of quality-of-life and adverse effects complements traditional measures of seizure control and helps guide individualized treatment strategies. Longitudinal studies will be important to determine whether reducing treatment-related symptom burden translates into sustained improvements in HRQOL.

## Data Availability

The data analyzed in this study is subject to the following licenses/restrictions: available on request from corresponding author. Requests to access these datasets should be directed to ab29@aub.edu.lb.
