# Supplementary material for: Determinants of health-related quality of life in pediatric epilepsy: the impact of treatment tolerability and epilepsy type
Source: Front Neurol. 2026 Aug 10;17:1893873. doi: 10.3389/fneur.2026.1893873 (PMC13500616; doi:10.3389/fneur.2026.1893873)
Supplement: Supplementary file 1 [file Table_1.DOCX]

**Supplementary Table 1 Hierarchical multivariable regression analyses of factors associated with QOLCE-55 subdomain scores**

|  | **Cognitive domain score** | | |  | **Physical domain score** | | |  | **Social domain score** | | |  | **Emotional domain score** | | |
| --- | --- | --- | --- | --- | --- | --- | --- | --- | --- | --- | --- | --- | --- | --- | --- |
|  | Model 1 | Model 2 | Model 3 |  | Model 1 | Model 2 | Model 3 |  | Model 1 | Model 2 | Model 3 |  | Model 1 | Model 2 | Model 3 |
|  | B | B | B |  | B | B | B |  | B | B | B |  | B | B | B |
| **Epilepsy duration (years)** | **-1,820*** | **-1,525*** | -1,407 |  | **-2,041*** | **-1,922*** | **-1,827*** |  | -0,756 | -0,466 | -0,403 |  | 0,915 | **1,124*** | 1,099 |
| **Time since last seizure (months)** | **0,548**** | 0,267 | 0,246 |  | **0,437*** | **0,323*** | 0,305 |  | **0,341*** | 0,065 | 0,053 |  | 0,250 | 0,051 | 0,055 |
| **Epilepsy group** |  |  |  |  |  |  |  |  |  |  |  |  |  |  |  |
| GGE vs SeLFEs | 3,253 | 4,375 | 4,179 |  | 4,931 | 5,386 | 5,228 |  | -2,367 | -1,262 | -1,367 |  | -3,806 | -3,008 | -2,966 |
| Focal Epilepsy vs SeLFEs | -5,079 | -4,106 | -3,638 |  | -2,945 | -2,550 | -2,172 |  | -9,661 | **-8,703*** | **-8,452*** |  | **-9,438*** | **-8,746*** | **-8,846*** |
| **Seizure Frequency/past three months** |  |  |  |  |  |  |  |  |  |  |  |  |  |  |  |
| None | Ref | Ref | Ref |  | Ref | Ref | Ref |  | Ref | Ref | Ref |  | Ref | Ref | Ref |
| Less than one per month | 18,274 | 18,016 | 18,387 |  | 5,878 | 5,773 | 6,073 |  | 6,174 | 5,920 | 6,119 |  | -2,902 | -3,086 | -3,166 |
| 1 – 3 seizures per month | -2,278 | 6,996 | 7,110 |  | 1,140 | 4,903 | 4,995 |  | **-13,400*** | -4,267 | -4,206 |  | **-11,853*** | -5,259 | -5,283 |
| 1 – 3 seizures per week | -11,436 | 3,407 | 2,725 |  | -13,552 | -7,528 | -8,078 |  | -15,284 | -0,667 | -1,032 |  | **-20,038*** | -9,485 | -9,339 |
| Daily | -11,991 | -0,893 | -3,303 |  | -4,997 | -0,493 | -2,439 |  | -11,460 | -0,531 | -1,823 |  | -1,054 | 6,838 | 7,354 |
| **LAEP Score** |  | **-1,312**** | **-1,280**** |  |  | **-0,532*** | **-0,506*** |  |  | **-1,292**** | **-1,275**** |  |  | **-0,933**** | **-0,940**** |
| **polytherapy vs. monotherapy** |  |  | -6,660 |  |  |  | -5,377 |  |  |  | -3,569 |  |  |  | 1,425 |

B: unstandardized Beta coefficient, LAEP: Liverpool Adverse Events Profile. GGE: Genetic generalized epilepsy**,** SeLFE: Self-limited focal epilepsy.

*p˂0.05, **p˂0.001

Model 1: clinical variables
Model 2: Model 1 + LAEP score
Model 3: Model 2 + treatment regimen
